# Assessment of Mutagenic Effect of *G. acerosa* and *S. wightii* in *S. typhimurium* (TA 98, TA 100, and TA 1538 strains) and Evaluation of Their Cytotoxic and Genotoxic Effect in Human Mononuclear Cells: A Non-Clinical Study

**DOI:** 10.1155/2014/313942

**Published:** 2014-05-20

**Authors:** Arif Nisha Syad, Pandima Devi Kasi

**Affiliations:** Department of Biotechnology, Alagappa University (Science Campus), Karaikudi, Tamil Nadu 630 004, India

## Abstract

The marine red algae (*Gelidiella acerosa* and *Sargassum wightii*) possessing excellent antioxidant and anticholinesterase activity were subjected to toxicity evaluation for a deeper understanding of other bioprotective properties of seaweeds. Cytotoxic evaluation was done by trypan blue exclusion, and MTT (3-(4,5-dimethylthiazol-2-yl)-2,5-diphenyltetrazolium bromide) assays using human PBMC (peripheral blood mononuclear cells) and RBC (red blood cells) lysis assay using human erythrocytes. Mutagenicity of the seaweeds was analyzed by Ames salmonella mutagenicity test with the histidine dependent mutant strains TA 98, TA100 and TA 1538. Genotoxic activity was verified in PBMC by comet assay. The results suggest that benzene extract of *G. acerosa* (BEGA) and dichloromethane extract of *S. wightii* (DMESW) did not show cytotoxic effect both in PBMC and erythrocytes. Evaluation of mutagenic activity suggests that the seaweeds did not cause any mutagenic effects both in the absence and the presence of S9 microsomal fraction in all the three *Salmonella* mutant strains. Results of genotoxic study showed that PBMC treated with seaweed extracts (1 mg/mL) exhibit less or no damage to cells, thus proving the non-genotoxic effect of the extract. Since these *in vitro* non-clinical studies clearly demonstrate the non-toxic nature of the seaweeds, they could be exploited for further characterization, which would result in development of novel and safe therapeutic entities.

## 1. Introduction

The exploitation of plants and other natural products as medicines has been in practice for several decades, since these natural products have excellent therapeutic potentials and serve as leads for the development of novel drugs [1, 2]. More than 80% of the population in the world use botanical preparations as medicines [3]. During the past 20 years, thousands of novel compounds with diverse biological activities ranging from antiviral to anticancer have been isolated from various marine sources [4]. Seaweeds or macroalgae are found to have rich source of secondary metabolites like polysaccharides, sterols, terpenoids, flavonoids, and fatty acids, which acts as excellent drug leads and facilitates the unraveling of novel biosynthetic pathways [5, 6]. However, certain metabolites are toxic in nature, which necessitates the toxicological evaluation of natural products, which includes testing of plants for cytotoxic, genotoxic, and mutagenic potentials [7, 8]. Mutagenicity is the process of induction of permanent transmissible changes in the genetic material of the cells, whereas genotoxicity is a broader term, which is the ability of a compound to interact with the genetic material and also with other cellular apparatus that maintains the fidelity of the genome [9]. Only non-toxic and non-mutagenic plants could be considered for relative safe use and also for detailed study of other pharmacological potentials [8].

The marine macroalgae* Gelidiella acerosa* and* Sargassum wightii* belong to the class of Rhodophyceae and Phaeophyceae, respectively.* G. acerosa* has been recognized as excellent source for superior quality of agar production [10]. Earlier reports have demonstrated that the sulfonoglycolipids and sphingosine derivatives obtained from* G. acerosa* were found to enhance the sperm motility and possess antiprogesterone activity, respectively [11]. However, very few studies are being carried out on other therapeutic potentials of* G. acerosa*. A wide range of* Sargassum* species has been reported to exhibit prominent antiviral and anticancer activities [12]. In addition to that, several reports have demonstrated the antioxidant and anticholinesterase activities of* S. sagamianum* (farnesylacetone derivatives),* S. thunbergii*,* S. polycystum* (polyunsaturated fatty acids), and* S. siliquastrum* (chromene) [13–15]. Recently, it has also been suggested that alginic acid derived from* S. wightii* exhibits anti-inflammatory effects in rats with arthritis [16]. Previous studies from our group have demonstrated that the benzene extract of* G. acerosa* (BEGA) possesses significant antioxidant [17] and anti-cholinesterase activities [18] when compared to other solvent extracts. Similarly, the dichloromethane extract of* S. wightii* (DMESW) has been found to possess excellent antioxidant and cholinesterase inhibitory activities [19]. The presence of high amount of terpenoids has been attributed to the above mentioned activities in both extracts. Apart from these therapeutic potentials, our study also demonstrates that both seaweeds possess high nutritional values [20]. Therefore, it is necessary to evaluate the toxicity profiles of* G. acerosa* and* S. wightii* to further evaluate other bioprotective properties. Hence the present study aims at evaluating the cytotoxicity, mutagenicity, and genotoxicity of BEGA and DMESW under* in vitro* condition.

## 2. Materials and Methods

### 2.1. Chemicals and Strains

MTT and sodium ammonium phosphate were purchased from Sigma Aldrich, USA. Glucose-6-phosphate, NADP (nicotinamide adenine dinucleotide phosphate), and LSM (lymphocyte separation medium) were obtained from HiMedia laboratories, India.* Salmonella typhimurium* strains TA98 (MTCC 1251), TA100 (MTCC 1252), and TA1538 (MTCC 1253) were purchased from Microbial Type Culture Collection (MTCC), India. All the other chemicals and reagents used were of analytical grade with the highest purity.

### 2.2. Collection of Seaweed Samples

Seaweeds (*Gelidiella acerosa* and* Sargassum wightii*) were collected from intertidal region in Gulf of Mannar and identified according to Oza and Zaidu [21] and Krishnamurthy and Joshi [22] and further confirmed by Dr. M. Ganesan, Scientist, CSMCRI, Mandapam Camp, Tamil Nadu. The voucher specimen was deposited at Department of Biotechnology, Alagappa University, under the accession number AUDBTGA20100101 and AUDBTSW20100102 for* G. acerosa* and* S. wightii*, respectively.

### 2.3. Processing of Seaweeds

The collected seaweeds were processed to remove the attached specimens on their surface. The samples were washed in tap water and in distilled water. To remove the adhered microflora, the seaweeds were washed with alcohol. The processed seaweeds were stored in airtight zip-lock containers and stored.

### 2.4. Extraction of Seaweeds

100 g of seaweeds (*Gelidiella acerosa* and* Sargassum wightii*) was packed in soxhlet apparatus. Around 300 mL of the respective solvent was placed in the solvent reservoir and the extraction was carried out for 6 h using a wide range of solvents successively (for instance, 100 g of seaweed was extracted with petroleum ether for 6 h; then the extract was collected and the same seaweed was used for extraction with hexane for 6 h and so on) in the following order: petroleum ether, hexane, benzene, dichloromethane, chloroform, ethyl acetate, acetone, methanol, and water. All the extracts were then subjected to redistillation to remove the solvents from the extracts. The extract was then filtered using Whatman number 1 filter paper and kept in a dessicator to remove the solvents completely. After drying, the extracts were weighed and stored until use for analysis. The extract was filter sterilized through syringe filters using cellulose acetate membrane (0.22 *μ*m) and the filtered extract was stored and used for further studies.

### 2.5. PBMC Isolation

Three mL of blood was collected from healthy volunteers in a tube containing EDTA (ethylene diamine tetra acetic acid), to which equal amount of LSM was added. Human PBMC (peripheral blood mononuclear cells) were separated by centrifugation at 2200 rpm for 15 minutes. The white layer formed intermittently was taken carefully and washed by using RPMI-1640 (Roswell Park Memorial Institute) medium at 2200 rpm for 15 minutes. The cell viability was assessed by trypan blue exclusion assay. PBMC were adjusted to 1 × 10^6^ cells/mL in complete medium and used for further experiments. The protocol was approved by the Institutional Ethics Committee of Alagappa University, Karaikudi, India (number IEC/ALU/1-2008)

### 2.6. Assessment of Cytotoxicity Using Trypan Blue Exclusion Assay and MTT Assay Using PBMC

PBMC were incubated with different concentrations (250, 500, and 1000 *μ*g/mL) of BEGA and DMESW at 37°C with 5% CO_2_ in water jacketed incubator for 24 h. The cell viability was checked at various time intervals (12 h, 18 h, and 24 h) and the loss of viability was compared with 1 mM H_2_O_2_. Trypan blue exclusion assay was performed according to Strober [23]. The stained and unstained cells were counted and the percentage of viability was calculated. MTT assay was performed according to the method of Mosmann [24] with slight modifications and the percentage of viability was calculated using the following formula:

(1) $$\begin{array}{c} \text{Cell}\;\;\text{survival}=\frac{\text{Mean}\;\;\text{absorbance}\;\;\text{in}\;\;\text{test}\;\;\text{wells}}{\text{Mean}\;\;\text{absorbance}\;\;\text{in}\;\;\text{control}\;\;\text{wells}}\times 100. \end{array}$$

### 2.7. Hemolysis Assay

The hemolysis assay was performed according to Fischer et al. [25] with minor modifications. Blood was taken from healthy volunteers and RBC were freshly isolated from the blood. The cells were washed thrice with freshly prepared 150 mM NaCl. After centrifugation at 2500 rpm for 15 min, the supernatant was removed and the cells were resuspended in 100 mM sodium phosphate buffer. 200 *μ*L of RBC solution was mixed with different concentrations of seaweed extracts (250, 500, and 1000 *μ*g/mL) and was made to equal 1 mL with phosphate buffer. Triton X-100 and sodium phosphate buffer (with milli Q water) were used as positive and negative controls, respectively. Then the tubes were incubated at 37°C in a water bath for 30 min. After incubation, the cells were centrifuged at 2500 rpm for 15 min. The amount of hemoglobin released was taken as a measure of cell lysis. The supernatant was collected and the absorbance was measured at 540 nm in a UV-Vis spectrophotometer.

### 2.8. Bacterial Reverse Mutation Test [26]

Bacterial reverse mutation test (Ames test) was performed to evaluate the mutagenic properties of the seaweeds. This test involves the use of histidine dependent bacteria that can grow on glucose minimal agar plate containing trace amount of histidine. The cells that revert to histidine independent (*his* ^+^) will be able to form colonies. The small amount of histidine allows all the plated bacteria to undergo a few cell divisions; in many cases, this growth is essential for mutagenesis to occur. The number of spontaneously induced revertant colonies is relatively constant for each strain. However, when a mutagen is added to the plate, the number of revertant colonies per plate will be increased in a dose related manner [26]. Interestingly, certain mutagens remain inactive unless they get metabolized into active forms. Unfortunately, the bacterial strains that are used for Ames test do not possess these systems and, hence, the oxidation system has to be introduced externally along with the substance, which is suspected. In order to achieve this, S9 microsomal fraction (9000 g supernatant fraction of rat liver homogenate), which is the rodent metabolic activation system, has been employed in the presence of cofactors such as NADP and NADPH [26].

A single colony of* Salmonella typhimurium* tester strains (TA 98, TA 100, and TA1538) was picked and inoculated into 3 mL of LB broth containing 0.5 mM Biotin and 0.05 mM Histidine and it was incubated at 37°C for 12 h. The culture was then centrifuged at 6600 rpm for 15 minutes at 4°C and the pellet obtained was resuspended in 1 mL of phosphate buffer. Incubation mixture was prepared as follows: 50 *μ*L of 0.015 M phosphate buffer mixed with different concentrations of seaweed extract (250, 500, 1000, 2000, and 4000 *μ*g/plate) and 50 *μ*L of bacterial suspension. The mixture was then incubated for 90 min at 37°C. 2.5 mL of molten surface agar containing 0.6% agar, 0.6% NaCl, 0.05 mM L-Histidine, and 0.05 mM Biotin was mixed with the incubation mixture and then poured over the glucose minimal agar plates. Sodium azide, a strong mutagen (1 *μ*g/plate), was used as positive control for the tester strain TA 100 and 4-nitroquinoline (0.1 *μ*g/plate) was used as positive control for the strains TA 98 and TA 1538. The plates were incubated for 66 h at 37°C.

The mutagenicity ratio (MR) was calculated as the number of revertants in the treated sample to the number of spontaneous revertants. If the value of MR is higher than 2.0, then the sample was considered as mutagenic.

### 2.9. Preparation of Rat Liver S9 Fraction and Mix

Healthy male Sprague-Dawley rats were treated with 30 mg/kg sodium phenobarbitone (in 0.9% w/v saline) on day one and 60 mg/kg on days 2, 3, and 4. After five days, animals were sacrificed by cervical dislocation and the liver was collected and homogenized in 0.15 M KCl. The homogenate was centrifuged at 9,000 g for 10 min and the supernatant was aliquoted (2 mL portions) and stored at −80°C until use. The S9 mix prepared includes 8 mM MgCl_2_, 33 mM KCl, 5 mM glucose-6-phosphate, 4 mM NADP, 0.1 M sodium phosphate buffer (pH 7.4), and 10% S9 fraction (v/v) [26].

### 2.10. Bacterial Reverse Mutation Test with Rat Liver S9 Microsomal Fraction

The procedure was similar to that of Ames test described above, but in addition to the components of incubation mixture, S9 mix was added. A single colony of* Salmonella typhimurium* tester strains was picked and inoculated into 3 mL of LB broth containing 0.5 mM Biotin and 0.05 mM Histidine and it was incubated at 37°C for 12 h. The culture was then centrifuged at 6600 rpm for 15 minutes at 4°C and the pellet obtained was resuspended in 1 mL of phosphate buffer. Incubation mixture was prepared as follows: 500 *μ*L of S9 mix (8 mM MgCl_2_, 33 mM KCl, 5 mM glucose-6-phosphate, 4 mM NADP, 100 mM sodium phosphate, and 10% S9 fraction), 50 *μ*L of 0.015 M phosphate buffer mixed with different concentrations of seaweed extract (250, 500, 1000, 2000, and 4000 *μ*g/plate), and 50 *μ*L of bacterial suspension. The mixture was then incubated for 30 min at 37°C. 2.5 mL of molten surface agar containing 0.6% agar, 0.6% NaCl, 0.05 mM L-Histidine, and 0.05 mM Biotin was mixed with the incubation mixture and then poured over the glucose minimal agar plates. 2-Aminoanthracene (2-AA) (1 *μ*g/plate) was used as positive control. The results were expressed as ratio of mutagenicity and the calculation was done as mentioned earlier.

### 2.11. Comet Assay

The genotoxic potential of BEGA and DMESW was assessed by comet assay/single gel electrophoresis assay according to the method of Singh et al. [27] with slight modifications. It is a rapid and a cell based method widely employed to detect various genotoxic agents. In this assay, the embedded cells in agarose gel were subjected to alkaline lysis and the extent of DNA damage caused by the suspected genotoxic agents was determined by measuring the tail moment, olive moment, and percentage of DNA in tail. PBMC suspension of >95% viability was adjusted to a concentration of 1 × 10^6^ cells/mL with complete medium and incubated for 24 h with various concentrations of extracts (250, 500, and 1000 *μ*g/mL). 250 *μ*M of H_2_O_2_ was used as positive control. After exposure the cells were centrifuged at 2200 rpm for 5 min and washed with PBS twice. The pellet was suspended in 0.75% low melting agarose (LMA) and poured over the slides which were precoated with normal melting agarose (NMA). The gel was covered with a glass cover slip and left to set at 4°C for 5 to 10 min. Gel embedded cells were lysed in lysis buffer for 12 h at 4°C. Electrophoresis was carried out in freshly prepared precooled buffer for 20 min at 25V. The slides were washed thoroughly with neutralization buffer and were stained with 10 *μ*L of ethidium bromide (10 *μ*g/mL) and viewed under confocal laser scanning microscopy. The scanned images were analyzed in Comet Score 1.5 software (TriTek's Revolutionary Technology). Around 100 cells were scored for each individual sample. The parameters employed for evaluating DNA damage were tail moment, olive moment, and percentage DNA in tail.

### 2.12. Statistical Analysis

Experimental results concerning this study were represented as mean ± S.D of three parallel measurements. All the statistical analysis was done using the software SPSS Statistics 17.0. The results of the cytotoxicity and mutagenicity tests were analyzed by one-way ANOVA. Mann-Whitney *U* test was performed to analyze the data in comet assay. *P* value < 0.05 was regarded as significant.

## 3. Results 

### 3.1. Assessment of Cytotoxic Effects of* G. acerosa* and* S. wightii* in Human Mononuclear Cells

**Figure 1:**
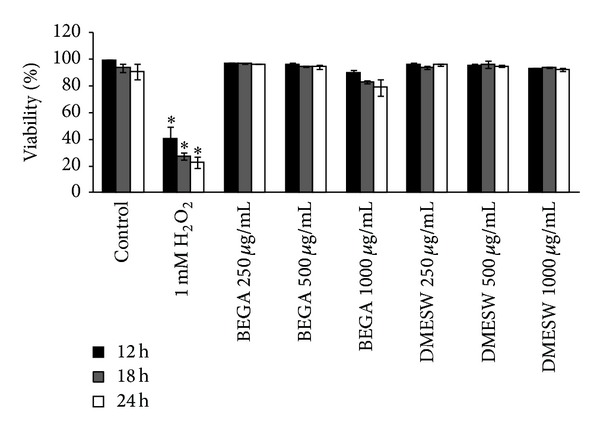
Cytotoxic evaluation of benzene extract of* G. acerosa* (BEGA) and dichloromethane extract of* S. wightii* (DMESW) by trypan blue exclusion assay. The values are expressed as mean ± S.D.

The preliminary cytotoxic evaluation of seaweed extracts was done by trypan blue exclusion assay, in which the effect of seaweed extracts on cell viability was evaluated at 12 h, 18 h, and 24 h. BEGA did not affect the cell viability at the concentrations of 250 and 500 *μ*g/mL. However, a slight decrease in the percentage of viability (90.23 ± 1.67, 82.79 ± 1.23, and 78.88 ± 5.97% at 12 h, 18 h, and 24 h, resp.) was observed at the concentration of 1 mg/mL (not statistically significant). Treatment with 1 mM H_2_O_2_ showed a significant decrease (*P* < 0.05) in cell viability (40.61 ± 8.48, 27.47 ± 2.82, and 22.78 ± 4.56% at 12 h, 18 h, and 24 h, resp.) (Figure 1). Similarly, the cytotoxic effect of DMESW was evaluated and the results suggest that DMESW also did not exhibit any cytotoxic effects when checked with various concentrations of 250 (95.97 ± 0.65%), 500 (94.87 ± 0.69%), and 1000 *μ*g/mL (92.33 ± 1.35%) in PBMC, even after incubating at 24 h.

**Figure 2:**
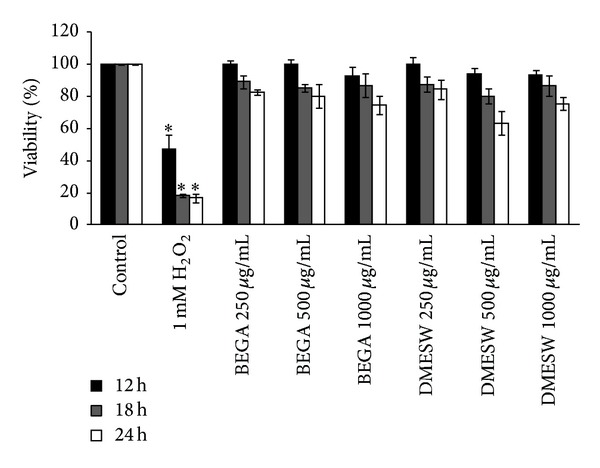
Evaluation of cytotoxicity of benzene extract of* G. acerosa* (BEGA) and dichloromethane extract of* S. wightii* (DMESW) by MTT assay. The values are expressed as mean ± S.D.

To further validate the non-cytotoxic effects of the seaweed extracts, MTT assay was performed. Treatment with 1 mg/mL of the extract for 12 h exhibited the percentage of viability of 92.78 ± 5.36 and 93.07 ± 3.1% for BEGA and DMESW, respectively (Figure 2). However, a slight decrease (not statistically significant) in the viability was observed when treated with increasing concentrations of both extracts, when incubated for 18 h and 24 h. 1 mM H_2_O_2_ was used as positive control and it reduces the cell viability significantly (*P* < 0.05) to 46.83 ± 9.41, 18.43 ± 0.79, and 16.75 ± 2.45% after incubation at 12 h, 18 h, and 24 h, respectively (Figure 2). The results suggest that both BEGA and DMESW were less cytotoxic in PBMC.

### 3.2. Evaluation of Membrane Disintegrating Effects of* G. acerosa* and* S. wightii* in Human Erythrocytes

**Figure 3:**
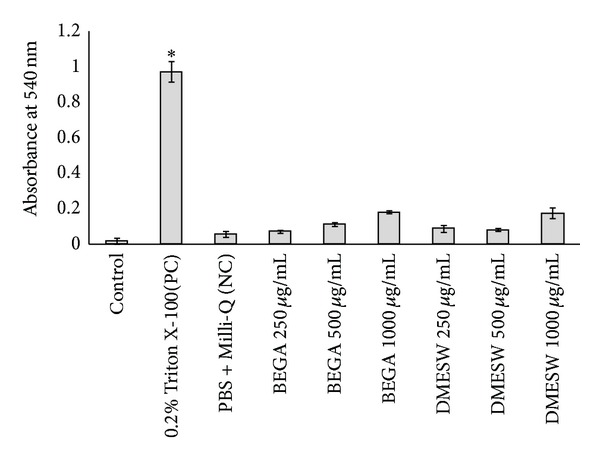
Determination of hemolytic activity of benzene extract of* G. acerosa* (BEGA) and dichloromethane extract of* S. wightii* (DMESW). The values are expressed as mean ± S.D.

Human erythrocytes were used for the evaluation of membrane disintegrating activity of seaweed extracts. RBC treated with various concentrations of BEGA exhibited the least absorbance of 0.071 ± 0.009 and 0.111 ± 0.013 for 250 and 500 *μ*g/mL, respectively. However, at 1 mg/mL, the BEGA induces a slight damage and exhibits the absorbance of 0.18 ± 0.01. A complete loss of membrane integrity was observed in the cells treated with 0.2% Triton X-100, as the absorbance at 540 nm was significantly high (*P* < 0.05) and increased to 1.40 ± 0.05 (Figure 3). In case of* S. wightii* the absorbance observed for 250, 500, and 1000 *μ*g/mL (0.089 ± 0.019, 0.080 ± 0.008, and 0.17 ± 0.03 resp.) was low (Figure 3). Therefore, the results suggest that both the BEGA and the DMESW did not interrupt or damage the erythrocyte membrane.

### 3.3. Assessment of Mutagenic Properties of* G. acerosa* and* S. wightii* Using* Salmonella typhimurium* Mutant Strains

The present study reveals that both the BEGA and the DMESW did not produce any mutagenic effect in all the three strains employed. At the concentration of 4 mg/mL of BEGA, the mutagenicity ratio (MR) exhibited by the tester strains of TA 98, TA 100, and TA 1538 was 0.5 ± 0.03, 0.82 ± 0.18, and 1.20 ± 0.17, respectively (Table 1). In the case of strains treated with positive controls (sodium azide for TA 100 and 4-nitroquinoline for TA 98 and TA 1538), a significant increase (*P* < 0.05) in MR was observed. The MR values obtained were 2.81 ± 0.26, 3.62 ± 0.53, and 11.31 ± 1.23 for the tester strains TA 98, TA 100, and TA 1538, respectively. Similarly in the case of 4 mg/mL of DMESW, very low MR values of 0.60 ± 0.07, 1.05 ± 0.002, and 0.84 ± 0.39, respectively, were exhibited for TA 98, TA 100, and TA 1538 (Table 1).

In addition to the above mentioned test, the nonmutagenic potential of seaweed extracts was further validated by the bacterial reverse mutation test in the presence of metabolic activation systems, that is, in the presence of S9 microsomal fraction. The results show that, at the concentration of 4 mg/mL (BEGA), the tester strains TA 98, TA 100, and TA 1538 exhibited the MR of 1.06 ± 0.13, 0.93 ± 0.03, and 0.44 ± 0.03, respectively, whereas treatment with 2-AA significantly (*P* < 0.05) increases the frequency of* his* ^+^ revertants and exhibited higher MR of 5.8 ± 0.93, 2.11 ± 0.07, and 2.07 ± 0.08 for TA 98, TA 100, and TA 1538, respectively (Table 2). Similarly, results of the mutagenic effect of DMESW suggest that at 4 mg/mL concentration, the MR observed was 1.16 ± 0.07, 0.82 ± 0.44, and 0.85 ± 0.25, respectively (Table 2). Hence the results suggest that the BEGA and DMESW were non-mutagenic and do not induce gene mutations.

### 3.4. Genotoxic Evaluation of* G. acerosa* and* S. wightii* Using Human Peripheral Blood Mononuclear Cells

**Figure 4:**
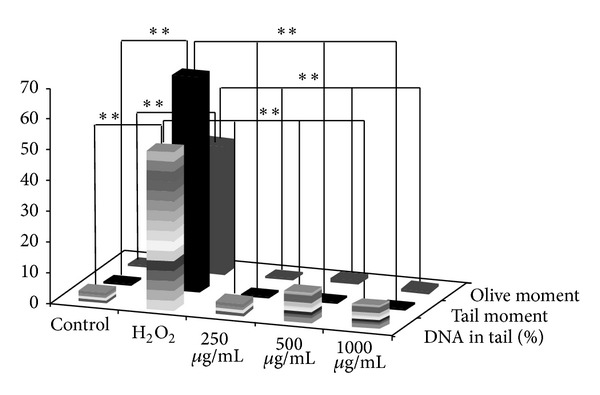
Assessment of genotoxicity of benzene extract of* G. acerosa* (BEGA) by comet assay.

**Figure 5:**
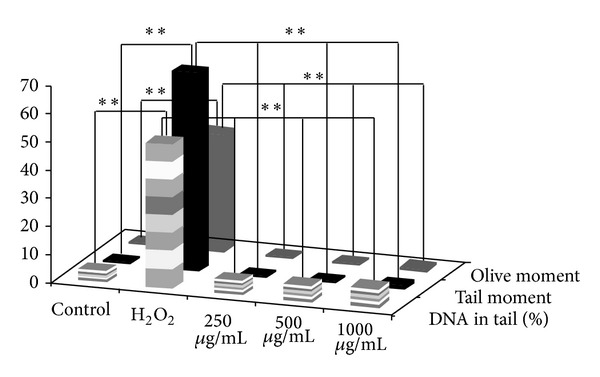
Assessment of genotoxicity of dichloromethane extract of* S. wightii* (DMESW) by comet assay.

Figure 4 illustrates the genotoxic effect of BEGA. The results show that the tail moment, olive moment, and percentage of DNA in tail were significantly high (*P* < 0.05) in the case of PBMC treated with 1 mM H_2_O_2_ when compared to the cells treated with seaweed extract. The tail moment, olive moment, and percentage of DNA in tail exhibited by the cells treated with H_2_O_2_ were 69.89, 42.15, and 51.38, respectively. Interestingly, the cells treated with 1000 *μ*g/mL of BEGA exhibited lesser tail moment, olive moment, and percentage of DNA in tail of about 1.22, 1.34, and 7.66, respectively. In the case of DMESW the tail moment, olive moment, and percentage of DNA in tail were very low (1.52, 1.58, and 7.26) at the concentration of 1000 *μ*g/mL (Figure 5). Therefore, the results suggest that both BEGA and DMESW do not induce any genotoxic effect in the PBMC.

## 4. Discussion

In the present study, the seaweeds* G. acerosa* and* S. wightii* were assessed for their cytotoxic, mutagenic, and genotoxic potentials. Preliminary assessment was made by employing trypan blue exclusion assay, where the extracts did not affect the viability of cells. In order to further validate the results, MTT assay was performed. MTT is a yellow dye which is reduced to purple colored formazan crystals by the activity of mitochondrial succinate dehydrogenase enzyme in viable cells. The increase in absorbance due to the formation of formazan crystals was measured at 540 nm, which corresponds to the viability of cells [8]. The results suggest that BEGA and DCMSW did not alter the cell viability except at the higher concentration, where a slight decrease in the viability of cells was observed. Recently, Choi et al. [28] also demonstrate a decrease in the viability of macrophages when treated with 400 *μ*g/mL of methanolic extract of another seaweed* Ecklonia cava*. A study led by Murugan and Iyer [29] showed growth inhibitory properties of* G. acerosa* and* S. wightii*, which was different from our results (non-cytotoxic potential). This could be due to the differences in the extraction methods and nature of extracts employed. For instance, in the present study, extraction was performed successively (not the whole crude extract) by hot extraction method, whereas in the former study, cold extraction method was employed and the growth inhibition assays were performed using crude (or whole) extract.

In spite of the fact that erythrocytes are simple blood cell type without any subcellular organelles, they can be exploited for testing* in vitro* toxicity of selected compounds by measuring the release of their hemoglobin content, which is generally represented as an index of cell membrane damage [30]. A significant increase in the absorbance was observed, when the cells were treated with 0.2% Triton X-100, which indicates a complete hemolysis pattern, whereas the cells treated with seaweed extracts did not show any increase in the absorbance at 540 nm, which suggests that both the BEGA and the DMESW did not interrupt or damage the erythrocyte membrane.

Ames* Salmonella* mutagenicity test has been recognized as the widely employed test to evaluate whether the given substance can produce genetic damage resulting in gene mutations. Kim et al. [31] show that the sporophyll of* Undaria pinnatifida* displayed no mutagenic effect in* S. typhimurium* strains irrespective of the presence or absence of S9 mix in the medium. A similar mutagenic evaluation was done on aqueous extract of the green alga* Spirogyra neglecta* by Thumvijit et al. [32] using* S. typhimurium *strains TA98 and TA100. The results show that the extract did not exhibit mutagenic effect both in the presence and the absence of S9 fraction. In the present study, the mutagenic properties of* G. acerosa* and* S. wightii* were evaluated using the histidine dependent* S. typhimurium* strains. The results of the test suggest that the extracts did not exhibit mutagenicity in all the three strains both in the presence and the absence of metabolic activation systems, in contrast to the standard mutagens, where a significant increase in the number of revertants was observed.

The assessment of genetic instability by DNA damage and repair is an important aspect of safety assessment of specific compounds. Comet assay is a simple and sensitive method of evaluating DNA damage in an individual cell. This assay can be performed in small number of cells and results can be obtained in a short period of time [33]. The degree of DNA damage can be represented using parameters like tail moment, olive moment, and percentage of DNA in tail. The brown seaweed* Fucus vesiculosus* was evaluated for genotoxic activity by Leite-Silva et al. [34] and the results suggest that the seaweed did not exhibit genotoxic effect in cultured human lymphocytes. Similarly the non-genotoxic property of* G. acerosa* and* S. wightii* was evaluated and the results show that the extracts did not induce DNA damage, which was verified by the decrease in the parameters like tail moment, olive moment, and percentage of DNA in tail.

## 5. Conclusion

Earlier studies indicate that the proposed seaweeds* G. acerosa* and* S. wightii* possess interesting antioxidant and ChE inhibitory activities. In addition to that, the nutritional composition of these seaweeds was also found to be high. Hence, in the present study, the safety aspects of* G. acerosa* and* S. wightii* were analyzed. The* in vitro* non-clinical toxicity study carried out in both seaweed extracts suggests that they were devoid of cytotoxic, mutagenic, and genotoxic effects. Therefore, these seaweeds could be a safe and a potential source of therapeutic compounds with excellent pharmacological activities. Further research is in progress to evaluate the toxicity profiles in rodents (Swiss albino mice) and to explore the active principles present in seaweeds (purification and characterization), which could be a productive approach in the facet of drug discovery and development.

## Figures and Tables

**Table 1:** Mutagenic evaluation of BEGA and DMESW by Ames test using *Salmonella typhimurium *tester strains TA 98, TA 100, and TA 1538.

Treatment	Concentration (µg/plate)	MR^a^ (TA 98)	MR^a^ (TA 100)	MR^a^ (TA 1538)
BEGA	250	1.24 ± 0.24	0.97 ± 0.12	0.81 ± 0.20
	500	1.21 ± 0.12	0.92 ± 0.07	0.81 ± 0.17
	1000	0.91 ± 0.27	0.94 ± 0.10	0.91 ± 0.45
	2000	0.5 ± 0.24	1.01 ± 0.03	1.13 ± 0.27
	4000	0.5 ± 0.03	0.82 ± 0.18	1.20 ± 0.17

DMESW	250	1.19 ± 0.35	0.97 ± 0.042	0.81 ± 0.16
	500	1.21 ± 0.31	1.19 ± 0.007	1 ± 0.23
	1000	0.56 ± 0.15	1.10 ± 0.35	0.63 ± 0.13
	2000	0.67 ± 0.29	1.00 ± 0.007	0.44 ± 0.12
	4000	0.60 ± 0.07	1.05 ± 0.002	0.84 ± 0.39

Negative control (distilled water)	40 µL	0.64 ± 0.19	0.87 ± 0.02	0.34 ± 0.07

Positive control	SA—1 µg/plate 4-NQ—0.11 µg/plate	2.81 ± 0.26**	3.62 ± 0.53**	11.31 ± 1.23**

^a^Results were expressed as mean ± SD (*n* = 3). MR: mutagenicity ratio; SA: sodium azide (standard mutagen for TA 100); 4-NQ: 4-nitroquinoline (standard mutagen for TA 98 & TA 1538);

BEGA: benzene extract of *G. acerosa.*

DMESW: dichloromethane extract of *S. wightii*.

**P < 0.05.

**Table 2:** Mutagenic evaluation of BEGA and DMESW by Ames test using *Salmonella typhimurium *tester strains with rat liver S9 microsomal fraction.

Treatment	Concentration (µg/plate)	MR^a^ (TA 98)	MR^a^ (TA 100)	MR^a^ (TA 1538)
BEGA	250	0.80 ± 0.13	0.78 ± 0.06	0.024 ± 0.005
	500	0.48 ± 0.13	0.64 ± 0.35	0.030 ± 0.008
	1000	0.64 ± 0.25	0.82 ± 0.04	0.041 ± 0.027
	2000	0.91 ± 0.10	0.55 ± 0.05	0.30 ± 0.008
	4000	1.06 ± 0.13	0.93 ± 0.03	0.44 ± 0.03

DMESW	250	0.04 ± 0.026	0.53 ± 0.28	0.98 ± 0.64
	500	0.33 ± 0.13	1.75 ± 0.43	0.74 ± 0.21
	1000	0.18 ± 0.04	1.68 ± 0.20	1.04 ± 0.08
	2000	0.97 ± 0.069	1.94 ± 0.49	0.80 ± 0.17
	4000	1.16 ± 0.07	0.82 ± 0.44	0.85 ± 0.25

Negative control (distilled water)	40 µL	0.28 ± 0.10	0.80 ± 0.05	0.036 ± 0.005

Positive control	2-AA—1 µg/plate	5.8 ± 0.93**	2.11 ± 0.07**	2.07 ± 0.08**

^a^Results were expressed as mean ± SD (*n* = 3).

MR: mutagenicity ratio; 2-AA: 2-aminoanthracene.

BEGA: benzene extract of *G. acerosa*.

DMESW: dichloromethane extract of *S. wightii*.

**P < 0.05.

## References

[1] Huang S-S, Huang G-J, Ho Y-L (2008). Antioxidant and antiproliferative activities of the four *Hydrocotyle* species from Taiwan. *Botanical Studies*.

[2] Castro LS, Perazzo FF, Maistro EL (2009). Genotoxicity testing of *Ambelania occidentalis* (Apocynaceae) leaf extract *in vivo*. *Genetics and Molecular Research*.

[3] Carabin IG, Burdock GA, Chatzidakis C (2000). Safety assessment of *Panax ginseng*. *International Journal of Toxicology*.

[4] Arif JM, Al-Hazzani AA, Kunhi M, Al-Khodairy F (2004). Novel marine compounds: anticancer or genotoxic?. *Journal of Biomedicine and Biotechnology*.

[5] Choi E-Y, Hwang H-J, Kim I-H, Nam T-J (2009). Protective effects of a polysaccharide from *Hizikia fusiformis* against ethanol toxicity in rats. *Food and Chemical Toxicology*.

[6] Lane AL, Mular L, Drenkard EJ (2010). Ecological leads for natural product discovery: novel sesquiterpene hydroquinones from the red macroalga *Peyssonnelia* sp. *Tetrahedron*.

[7] Verschaeve L, Van Staden J (2008). Mutagenic and antimutagenic properties of extracts from South African traditional medicinal plants. *Journal of Ethnopharmacology*.

[8] Varalakshmi KN, Sangeetha CG, Samee US, Irum G, Lakshmi H, Prachi SP (2011). *In vitro* safety assessment of the effect of five medicinal plants on human peripheral lymphocytes. *Tropical Journal of Pharmaceutical Research*.

[9] Maurici D, Aardema M, Corvi R Genotoxicity/mutagenicity. In: establishment of time tables for the phasing out of animal experiments for cosmetics. http://ec.europa.eu/consumers/sectors/cosmetics/files/doc/antest/(5)_chapter_3/7_genotox-mutagen_en.pdf.

[10] Prasad K, Siddhanta AK, Ganesan M, Ramavat BK, Jha B, Ghosh PK (2007). Agars of *Gelidiella acerosa* of west and southeast coasts of India. *Bioresource Technology*.

[11] Premakumara GAS, Ratnasooriya WD, Tillekeratne LMV, Amarasekare AS, Atta-Ur-Rahman A-U (2001). Human sperm motility stimulating activity of a sulfono glycolipid isolated from Sri Lankan marine red alga *Gelidiella acerosa*. *Asian Journal of Andrology*.

[12] Dias PF, Siqueira JM, Vendruscolo LF (2005). Antiangiogenic and antitumoral properties of a polysaccharide isolated from the seaweed *Sargassum stenophyllum*. *Cancer Chemotherapy and Pharmacology*.

[13] Ryu G, Park SH, Kim ES, Choi BW, Ryu SW, Lee BH (2003). Cholinesterase inhibitory activity of two farnesylacetone derivatives from the Brown Alga *Sargassum sagamianum*. *Archives of Pharmacal Research*.

[14] Kim J-A, Kong C-S, Kim S-K (2010). Effect of *Sargassum thunbergii* on ROS mediated oxidative damage and identification of polyunsaturated fatty acid components. *Food and Chemical Toxicology*.

[15] Jang KH, Lee BH, Choi BW, Lee H-S, Shin J (2005). Chromenes from the brown alga *Sargassum siliquastrum*. *Journal of Natural Products*.

[16] Lee JC, Hou MF, Huang HW (2013). Marine algal natural products with anti-oxidative, anti-inflammatory, and anti-cancer properties. *Cancer Cell International*.

[17] Suganthy N, Nisha SA, Pandian SK, Devi KP (2013). Evaluation of *Gelidiella acerosa*, the red algae inhabiting South Indian coastal area for antioxidant and metal chelating potential. *Biomedicine & Preventive Nutrition*.

[18] Syad AN, Shunmugiah KP, Kasi PD (2012). Assessment of anticholinesterase activity of *Gelidiella acerosa*: implications for its therapeutic potential against Alzheimer’s Disease. *Evidence-Based Complementary and Alternaternative Medicine*.

[19] Syad AN, Shunmugiah KP, Kasi PD (2013). Antioxidant and anti-cholinesterase activity of *Sargassum wightii*. *Pharmaceutical Biology*.

[20] Syad AN, Shunmugiah KP, Kasi PD (2013). Seaweeds as nutritional supplements: analysis of nutritional profile, physicochemical properties and proximate composition of *G. acerosa* and *S. wightii*. *Biomedicine & Preventive Nutrition*.

[21] Oza RM, Zaidu A (2003). *Revised Checklist of Indian Marine Algae*.

[22] Krishnamurthy V, Joshi HY (1970). *Check List of Indian Marine Algae*.

[23] Strober W (1997). Trypan blue exclusion test of cell viability. *Current Protocols in Immunology*.

[24] Mosmann T (1983). Rapid colorimetric assay for cellular growth and survival: application to proliferation and cytotoxicity assays. *Journal of Immunological Methods*.

[25] Fischer D, Li Y, Ahlemeyer B, Krieglstein J, Kissel T (2003). *In vitro* cytotoxicity testing of polycations: influence of polymer structure on cell viability and hemolysis. *Biomaterials*.

[26] Mortelmans K, Zeiger E (2000). The Ames Salmonella/microsome mutagenicity assay. *Mutation Research*.

[27] Singh NP, McCoy MT, Tice RR, Schneider EL (1988). A simple technique for quantitation of low levels of DNA damage in individual cells. *Experimental Cell Research*.

[28] Choi J-S, Bae H-J, Kim S-J, Choi IS (2011). *In vitro* antibacterial and anti-inflammatory properties of seaweed extracts against acne inducing bacteria, *Propionibacterium acnes*. *Journal of Environmental Biology*.

[29] Murugan K, Iyer VV (2013). Differential growth inhibition of cancer cell lines and antioxidant activity of extracts of red, brown, and green marine algae. *In Vitro Cellular & Developmental Biology—Animal*.

[30] Akanji MA, Ojewuyi OB, Yakubu MT (2013). Assessment of erythrocytes as a model for in vitro drug toxicity. *Fountain Journal of Natural and Applied Sciences*.

[31] Kim K-J, Lee O-H, Lee B-Y (2010). Genotoxicity studies on fucoidan from Sporophyll of *Undaria pinnatifida*. *Food and Chemical Toxicology*.

[32] Thumvijit T, Inboot W, Peerapornpisal Y, Amornlerdpison D, Wongpoomchai R (2013). The antimutagenic and antioxidant properties of *Spirogyra neglecta* (Hassall) Kützing. *Journal of Medicinal Plant Research*.

[33] Jaiswal M, Anuradha G, Rajeswari N, Ahuja YR (1995). Single cell gel electrophoresis assay (comet assay): its importance in human biology. *International Journal of Anthropology*.

[34] Leite-Silva C, Gusmão CLS, Takahashi CS (2007). Genotoxic and antigenotoxic effects of *Fucus vesiculosus* extract on cultured human lymphocytes using the chromosome aberration and Comet assays. *Genetics and Molecular Biology*.

